# Sympathetic Affections of the Eye

**Published:** 1871-10

**Authors:** 


### Sympathetic Affections of the Eye

Persons who have lost the use of one eye, either from accidental injuries, or from ulcerations of the cornea, the eye-ball becoming shrunken within the orbit, being at times painful and irritable, causing a sensitiveness to light in the sound eye, often become entirely blind for want of proper advice—not knowing what is necessary to be done to avert such a calamity.

An eye, the vision of which has been destroyed by particles of steel, stone, iron, or other foreign particles becoming imbedded in the cornea, or within the humors of the eye; if, after the inflammation resulting from the injury has subsided, it should again become sensitive to the touch, appearing inflamed or congested, discharging hot tears, and finally affecting the sound eye through sympathy,—such an eye is greatly endangering the patient to total blindness, and requires skillful management, at the hands of a competent surgeon.

If an eye that has become atrophied, presenting an appearance of a flat body no larger than a bean, and having caused no pain or inconvenience for years, should suddenly becom painful, discharging hot tears, and causing a unpleasant sensitiveness to light in the soun eye, the condition of affairs is such as to gi ample cause for alarm.

The patient and his friends are apt to attribute the symptoms to "cold in the eye," thinking that it will soon recover; and, neglecting to consult an oculist, the pain becomes worse, the well eye grows more and more sensitive to the light, with pain in the globe or over the orbit, until the symptoms are considered to be neuralgic, and liniments and lotions applied with a view of arresting the distress.

Soon, the inflammation will subside, the soreness leaves the blind eye, while the well one recovers its former usefulness. But, soon the patient is annoyed by little black dots, or spider webs, that seem to be floating before the vision. Sometimes they assume a more brilliant hue, appearing like sparks of fire, and again, like a monstrous spider, that is continually seen before the eye.

Occasionally the "cold" recurs in the b[illegible] eye, when it becomes painful and sens[illegible]tive t[illegible] the touch, while the hot tears again f[illegible]all fro[illegible] both eyes. These are the usual sym[illegible]ptoms th[illegible] accompany a shrunken and disea[illegible]sed eye, a[illegible] which are too often neglected [illegible]ntil the visi[illegible] in the remaining eye is also lo[illegible]

The history of thousands of cases, which have been reported in the large European Ophthalmic Hospitals, as well as by American Oculists, reveals the fact that there is but one course to be pursued, when the symptoms above described, manifest themselves. The remedy is one from which the patient always shrinks with alarm, but which, if properly considered by the patient, and fully explained by the surgeon, need cause no fear or anxiety for the result.

The only remedy is immediate extirpation of the blind eye, which can be quickly, safely, and painlessly done, under the influence of an anæsthetic.

We are sure that any person suffering from pain in a useless and disfigured eye, and one that, if allowed to remain, will certainly destroy vision in the sound one, would not hesitate to yield to the advice of a surgeon in whom they had confidence, and have the offending eye promptly removed.

Not the least danger accompanies such an operation, when in the hands of a skillful surgeon, and the relief afforded is instantaneous--the pain, hot tears, spots and webs, all disappear. The wound heals in three days' time, and, at the expiration of a month, the patient can wear an artificial eye, which, when carefully selected and accurately fitted, will bear close scrutiny, often defying detection. In the removal of the diseased eye, the muscles are left remaining, so that they form a stump over which the artificial eye fits, and by means of which it is caused to move almost as naturally as the opposite eye. In wearing, they give no pain whatever, but, on the contrary, protect the stump from atmospheric changes, and from the introduction of cinders and other foreign particles that would otherwise find a lodgment in the open, unprotected socket.

Therefore, let us again warn those who have a sound eye, suffering through sympathy, with an injured one—more especially one that has some foreign body lodged within the chamber,—to beware how they mistake this sympathetic inflammation for a mere "cold in the eye," for which they apply local applications, expecting so to cure the disease. It is a common error, and one that has consigned thousands of persons to hopeless, incurable blindness.

---

CARBOLIC acid, sprinkled in small quantities about, a room will abate those intolerable nuisances, fleas and mosquitoes.

